# Gender differences in a forensic psychiatric ward: A retrospective study

**DOI:** 10.1192/j.eurpsy.2021.344

**Published:** 2021-08-13

**Authors:** M. Jesus

**Affiliations:** Centro De Responsabilidade Integrada De Psiquiatria, Centro Hospitalar e Universitário de Coimbra, Coimbra, Portugal

**Keywords:** infanticide, Gender, mood disorders

## Abstract

**Introduction:**

The criminality associated with psychiatric disorders has been extensively studied with some studies showing a greater risk of violence in these patients. The gender differences in the general psychiatric population and can have an impact in the characteristics of a forensic population.

**Objectives:**

The authors aim to study the gender differences regarding diagnosis, type of crime and other characteristics in a forensic ward population.

**Methods:**

A retrospective study was designed, including patients admitted in the Forensic ward of Coimbra Hospital and University Center between 2018 and 2020.

**Results:**

Our study included 110 patients, 19 women and 91 men. Although psychotic disorders were the most common in both groups, particularly schizophrenia, mood disorders were significantly more common in women, with a risk of 7,768. This was explained by a greater prevalence of depressive episodes in women. These were associated with a particular type of crime, infanticide, that was not found in the men group. This might contribute to a greater prevalence of violent crimes in women. There was a chance of committing crimes against the offspring of 24 in women. The use of psychoactive substances was significantly greater in men, with a chance of 12,906.

**Conclusions:**

Considering that mood disorders are more common in women, these findings are easy to understand. The predominance of female perpetrators in infanticide is well described in the literature and can be associated with peripartum depression and gender roles. In this sample substance abuse was more common in man, like it’s seen in the general population.

**Disclosure:**

No significant relationships.

